# DNA Methylation Modulates Aging Process in Adipocytes

**DOI:** 10.14336/AD.2021.0904

**Published:** 2022-04-01

**Authors:** Hao Xie, Xin Liu, Qing Zhou, Teng Huang, Lu Zhang, Jia Gao, Yuhan Wang, Yanjun Liu, Tong Yan, Shu Zhang, Cong-Yi Wang

**Affiliations:** ^1^The Center for Biomedical Research, Department of Respiratory and Critical Care Medicine, NHC Key Laboratory of Respiratory Disease, Tongji Hospital, Tongji Medical College, Huazhong University of Science and Technology, Wuhan, China.; ^2^Department of Interventional Radiology, Renmin Hospital of Wuhan University, Wuhan, China.; ^3^The Center for Obesity and Metabolic Health, Affiliated Hospital of Southwest Jiaotong University, The Third People’s Hospital of Chengdu, Sichuan, China.; ^4^The Center of Gastrointestinal and Minimally Invasive Surgery, Department of General Surgery, The Third People’s Hospital of Chengdu & The affiliated Hospital of Southwest Jiaotong University, Chengdu, Sichuan, China.

**Keywords:** DNA methylation, aging, adipocytes, adipose tissue

## Abstract

Aging has been recognized to be a highly complex biological health problem with a high risk of chronic diseases, including type 2 diabetes, atherosclerosis, chronic bronchitis or emphysema, cancer and Alzheimer's disease. Particularly, age-related turnover in adipose tissue is a major contributor to metabolic syndromes and shortened lifespan. Adipocytes undergo senescence in early stage, which results in adipose tissue metabolic dysfunction, redistribution, and inflammation. The well-established association between DNA methylation (DNAm) and aging has been observed in the past few decades. Indeed, age-related alteration in DNAm is highly tissue-specific. This review intends to summarize the advancements how DNAm changes coupled with aging process in adipose tissue, by which DNAm regulates cellular senescence, metabolic function, adipokine secretion and beiging process in adipocytes. Elucidation of the effect of DNAm on adipose aging would have great potential to the development of epigenetic therapeutic strategies against aging-related diseases in clinical settings.

Adipose tissue (AT) was originally regarded as the largest energy storage organ in the body, but it has been subsequently recognized to be an endocrine organ capable of synthesizing a number of biologically active compounds that regulate metabolic homeostasis. Composed of mature adipocytes, luxuriant microvasculature and complicated stromal vascular fraction (SVF), the heterogeneous adipose tissue is roughly divided into two predominant types in mammals: white adipose tissue (WAT) and classical brown adipose tissue (BAT) [1]. Mature adipocytes derived from adipose progenitor cells are the main functional undertakers in adipose tissue. The SVF is composed of many different cell types, including adipose progenitor cells, endothelial cells, fibroblasts and immune cells. In response to energy demands, WAT including subcutaneous, visceral, omental and local-specific subtypes, plays a significant role in maintaining systemic energy homeostasis through the interplay between lipogenesis and lipolysis [2]. In the case of an urgent demand for energy, lipid mobilization is activated to breakdown stored triglycerides into fatty acids and glycerol [3]. Moreover, white adipocytes are capable of handling cytotoxic free fatty acids (FFAs) by converting them into less damaging neutral triglycerides, thereby protecting other tissues from lipotoxicity [4]. Once the capacity of lipid storage in AT is impaired (e.g., overnutrition or aging), lipids cannot be absorbed by the subcutaneous AT, which leads to ectopic lipid deposition [5, 6]. Age-related lipid deposition in the liver is associated with the development of fatty liver disease, while in the muscle it is correlated with insulin resistance and glucose intolerance [7, 8]. Additionally, BAT utilizes fatty acids and glucose for thermogenesis manifested by the overexpression of uncoupling protein-1 (UCP1) to generate heat [9, 10]. The unique non-shivering thermogenic property of brown adipocytes is activated under cold or sympathetic nerve-derived norepinephrine stimulation [11, 12]. Another type of adipocytes named beige or brite adipocytes emanate from smooth-muscle-like progenitors or white adipocytes [13, 14]. The cold-inducible or β-adrenergic activated beige adipocytes increase energy expenditure, similar to brown adipocytes, but exhibit a completely different developmental origin [15, 16]. Recent evidence demonstrates that cold-inducible beige fat biogenesis may be a potential therapy for obesity and type 2 diabetes [17-19].

Of note, AT plays a crucial regulatory role by releasing adipokines, such as adiponectin [20, 21], leptin [21, 22], interleukin-6 (IL-6) [23], IL-1β [24], IL-10 [25] and tumor necrosis factor-α (TNF-α) [26, 27]. In particular, adiponectin is primarily synthesized by white adipocytes to regulate insulin sensitivity and tissue inflammation [28]. IL-6 also promotes the release of free fatty acids (FFAs) from adipocytes, which impairs liver and pancreatic β-cell function [29, 30]. White adipocytes also secrete anti-inflammatory cytokine IL-10 to inhibit macrophage function and the production of pro-inflammatory cytokines in obesity [25, 31]. Similarly, BAT has also been found to secrete fibroblast growth factor-21 (FGF21), myostatin and C-X-C motif chemokine ligand-14 (CXCL14), thereby enhancing heat generation and regulating the functional properties of skeletal muscle and macrophages [32-34]. Ependymin-related protein 1 (EPDR1), a novel batokine secreted from human brown adipocytes, improves whole-body metabolism [35]. Therefore, AT is also an endocrine organ in addition to being energy reservoir. Unfortunately, aging occurs in AT even under 30 years old in humans as manifested by the redistribution of fat in the body [36, 37]. There is compelling evidence that its functionality is significantly impacted by the aging process, which is tightly controlled by multiple mechanisms, particularly by the epigenetic alterations such as the changes of DNA methylation (DNAm).

Indeed, DNAm has now been recognized as one of the major characteristic epigenetic alterations in mammalian cellular senescence [38, 39], which manifests a significant influence on the development and differentiation of stem cells or progenitor cells [40, 41]. DNAm is processed by five methyltransferases, DNMT1, DNMT2, DNMT3A, DNMT3B, and DNMT3L [42-45]. DNA methylome-encoded information is read by a family of methyl-CpG-binding domain (MBD) proteins, including MBD1, MBD2, MBD3, MBD4 and MeCP2 [43, 46-48]. In contrast, DNA demethylation is processed by two different pathways: one is the ten-eleven translocation enzymes (TETs) that oxidize the methylated base, and the other is activation induced deaminase (AID), which deaminates the methylated or a nearby base [49] (Table 1). In general, DNAm occurs at CpG sites, but a few do occur in non-CpG sites, which directly changes the accessibility of DNA to transcription factors and other DNA binding proteins [50]. Hypomethylated CpGs in the regulatory regions are generally correlated with constitutive upregulation of gene expression, while hypermethylated CpGs contribute to transcriptional repression [51]. In response to genetic inheritance and environmental insult, the dynamics of cellular methylation and demethylation jointly regulate gene expression and cell function relevant to aging process. Therefore, in this review we sought to update the advancement of DNAm, a major epigenetic mechanism, in the regulation of aging process in adipose tissues.

**Table 1:** Changes in DNA methyltransferases activity during aging.

Methyltransferases	Function	Activity	Reference
DNMT1	Maintenance methyltransferase	Decreased in fibroblasts, oligodendrocyte progenitor cells	[44,45]
DNMT2	Methylation of cytosine 38 in the anticodon loop of aspartic acid transfer RNA	Not clear	
DNMT3a/3b	De novo methylation	Increased in fibroblasts, oligodendrocyte progenitor cells	[44,45]
DNMT3L	Does not contain methyltransferase active site motifs but binds to the carboxyl-terminal domains of DNMT3a/3b	Not clear	

The relationship between DNA demethylases (TETs and AID) activity and aging is not clear. DNMT: DNA methyltransferase; TETs: ten-eleven translocation enzymes; AID: activation induced deaminase.

## Senescence induces redistribution and cellular dysfunction of adipose tissues

Cellular and molecular features of aging are associated with cellular senescence, dysregulation of nutritional sensing, and stem cell exhaustion [52]. Cellular senescence is also a defensive mechanism involved in embryogenesis and tumorigenic cell neutralization. Once senescent cell abundance exceeds a critical threshold, systemic dysfunction occurs. The lifelong aging process is initiated in all tissues over time, and particularly occurs far earlier in AT, as evidenced by the presence of facial wrinkles along with fat redistribution (e.g., skin fold, cheek sag, thin hands and legs) [53, 54]. A characteristic feature of fat redistribution during the aging process is the remarkable reduction in the subcutaneous AT and BAT along with an increase in visceral AT [55, 56]. For example, the presence of scapular BAT during infancy is gradually lost with age [57], and adolescent active BAT can only be detected in the supraclavicular region after cold exposure and becomes undetectable in individuals over the age of sixty [58-60].

In general, the mean white adipocyte size increases along with aging process, then decreases at the advanced aging stage (≧24 months in mice and ≧70 years in humans) [61, 62]. Similarly, brown adipocytes also manifest enlarged diameter and increased lipid droplet size with age in mice [63]. There is evidence that 3T3-L1 adipocytes volume gradually increases after induced maturation [64]. Moreover, aged mature adipocytes are characterized by the smaller dislocated nuclei and a large central lipid droplet (LD) surrounded by smaller ones [64]. Adipose tissues plasticity is impaired along with the progression of aging process as well. The ability of preadipocytes to replicate and differentiate into mature white or brown adipocytes declines with age, as manifested by the decrease of expression for adipogenic transcription factors PPAR-γ and C/EBP-α [65, 66]. Aging is associated with decreased lipid storage, fatty acid β-oxidation, and endocrine functions in white adipocyte. Moreover, the thermogenic capacity of BAT is reduced with age and coupled with downregulation of UCP1 expression as well [56]. Moreover, lipids are redistributed outside of the fat pool, accumulating in the liver, bone marrow, skeletal muscle, and other ectopic sites in old age. AT not only undergoes dramatic redistribution, but also couples with microenvironmental changes during the course of aging. In addition, mature adipocytes, progenitor cells, immune cells, and endothelial cells gradually senesce at different levels with aging in the heterogeneous and complex adipose tissue.

Senescent adipocytes exhibit a senescence-associated secretory phenotype (SASP) that includes a multitude of pro-inflammatory cytokines and chemokines triggering secondary senescence in neighboring cells [67]. Senescent cell accumulation in adipose tissue with advancing age results in both local and systemic dysfunction. P16-positive cells isolated from mouse inguinal adipose tissue exhibit high IL-6 expression and damage the function of adipocytes and impair insulin sensitivity in adipose tissue [66, 68]. Specifically, the accumulation of senescent preadipocytes and infiltrated immune cells, and increased pro-inflammatory cytokines perturb the microenvironment in AT, attenuating the lipid-handing capability of adipocytes, which then impairs adipogenesis and β-oxidation converying higher risk of FFA-mediated lipotoxicity [37, 69, 70]. As such, the irreducible FFA and inordinate profile of adipokines produced by senescent adipocytes in turn increase the infiltration of inflammatory macrophages and other immune cells [71-73]. Indeed, the percentage of macrophages is positively correlated with age in human omental AT [74, 75]. In addition to macrophages, accumulation of neutral killer (NK) cells, neutrophils, eosinophils, mast cells, dendritic cells, and lymphocytes in AT exacerbate immune competence by promoting inflammasome activation and formation of crown-like structures [76, 77]. Likewise, inappropriate adipokines derived from senescent adipocytes and infiltrated immune cells affect the production of insulin and glucagon from the pancreas, leading to high blood glucose and insulin resistance, central obesity, dyslipidemia, and hypertension [78].

**Figure 1.**
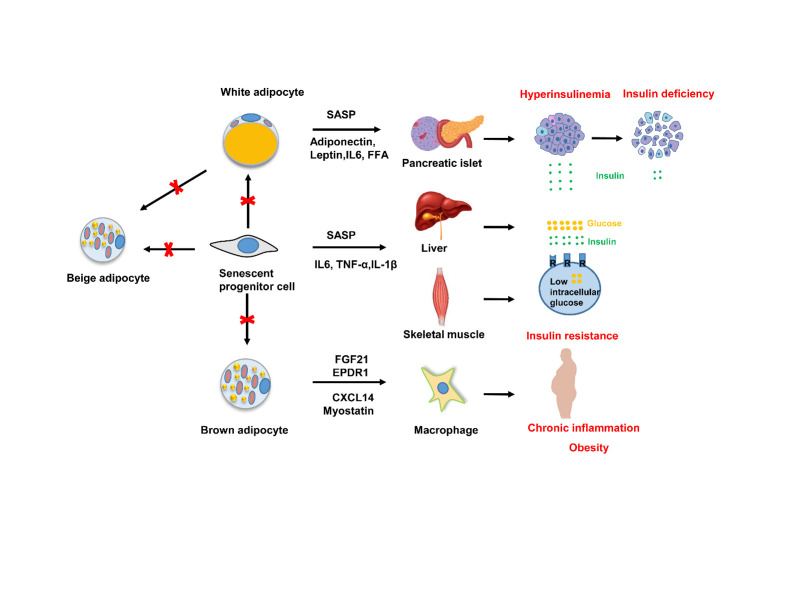
The dysfunction of adipose tissue during aging. Aging leads to the redistribution of adipose tissue and reduces the ability of adipose precursor cells to differentiate into mature adipocytes [153, 170]. The senescence-associated secretory phenotype (SASP) of senescent adipocytes impairs the function of pancreatic islet, liver and skeletal muscle, aggravating obesity and insulin resistance with age. Senescent adipocytes secrete a multitude of pro-inflammatory cytokines and chemokines triggering immune cell infiltration. Increased infiltrated cells and inflammatory factors (IL-6, IL-1β, TNF-α) [23, 24, 26], and decreased protective adipocytokines (adiponectin, leptin, FGF21, CXCL14) lead to systemic insulin resistance, and obesity [33, 102, 171].

More importantly, aging is associated with a decline in preadipocyte proliferation and differentiation, which then prevents adipogenesis and subsequent tissue replacement and regeneration [79-81]. In particular, adipose tissue-derived mesenchymal stem cells (AT-MSCs) from elderly subjects are featured by the increased expression of senescence-related genes and severely reduced beiging capability [82]. The loss of BAT mass coupled with reduced thermogenic capacity is caused by the conversion of brown into white-like adipocytes along with defective proliferation of brown adipogenic progenitor cells during aging [83, 84]. Interestingly, inhibition of the senescent signaling pathway p38/MAPK-p16Ink4a in adipose progenitor cells restores the beiging process and improves insulin resistance in aged individuals or mice [85]. In sharp contrast, activation of this particular pathway in young subjects accelerates preadipocyte senescence and prevents beiging induction [38]. Collectively, dramatic fat redistribution, irreducible lipotoxicity, excessive inflammation, and defective beiging are typical characteristics relevant to AT aging (Fig. 1).

## DNA methylation regulates the functional decline in aging white adipocytes

Upon the development of high-throughput technologies for the analysis of genome-wide DNAm levels, a large number of site-specific DNAm patterns had been characterized [52, 86]. Therefore, DNAm has now been recognized as having great potential as a biomarker for the aging process [87, 88]. Apparently, numerous studies have observed age-related methylation patterns associated with the function of adipocytes. However, it is still a challenge to identify more critical genes and pathways that are regulated by DNAm during the course of aging process in adipocytes before its application as a biomarker in clinical settings.

Over the past decade, studies have shown that DNAm comprises a key epigenetic machinery regulating the developmental processes and homeostasis of energy expenditure and storage in white adipocytes [89-91]. Increased methylation levels in the 3' untranslated region and genomes relative to the promoter region are observed in adipose tissue from obese women [92]. Even omental and subcutaneous adipose tissue show significant differential methylation profiles under the influence of external environment [92, 93]. Whole-genome DNAm analysis further confirms that the thrust of genes or genomic regional CpG DNAm pattern changes are noted in white adipocytes with aging [94, 95]. For example, methylation of the master adipogenic regulator *Ppar-γ* promoter region inhibits its transcription and translation, which contributes to the pathogenesis of metabolic complications during aging [96, 97]. Consistently, the adipogenic activity of white adipocytes declines with age in subcutaneous AT, coupled with a decreased level of PPAR-γ, leading to AT redistribution, and insulin resistance [98, 99]. Interestingly, obesity induces methylation of the *Ppar-γ* promoter in visceral AT from diabetic mice, causing a concomitant decrease in mRNA expression compared to that in normal mice [100]. In addition, the paracrine effects of senescent white adipocytes also trigger systemic inflammation, insulin resistance and senescence in the distally regulated organs.

**Figure 2.**
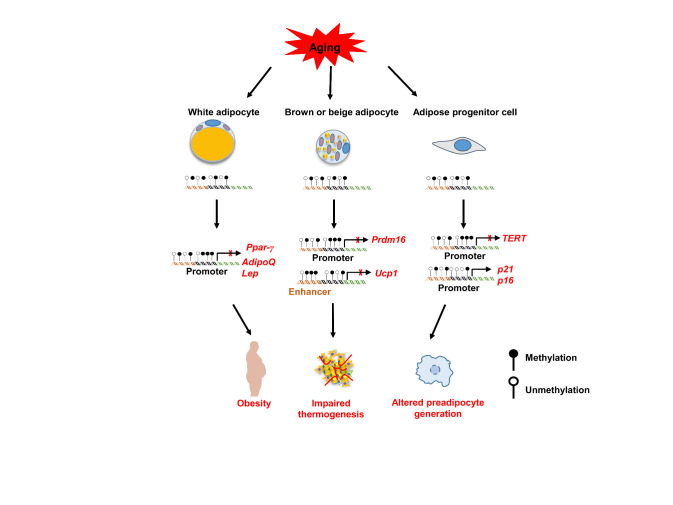
Effects of cellular DNA methylation on adipocytes senescence. Accumulated environmental factors and irreversible aging lead to changes in methylation patterns in adipocytes. Pathological increases in methylation levels of the promoter in *Ppar-γ* [100], *AdipoQ* [103], and *Lep* [114] result in white adipocyte glucose and lipid metabolism disorders, which further aggravate insulin resistance, obesity, and inflammation during aging. The promoter region of *PRDM16* and the enhancer region of *UCP1* tend to be hypermethylated during cellular senescence, leading to loss of beige adipose tissue and thermogenic properties of brown adipocytes [119, 123]. Impaired beige fat biogenesis and a transition from beige to white adipocytes positively correlate with fat inflammation and hyperinsulinemia. The hypomethylation of the promoter of senescence relevant genes (*p21*, *p16)* and hypermethylation of the *TERT* promoter accelerate adipose progenitor cellular senescence and exhaustion [172, 173].

Aging disrupts the secretion and synthesis of adipokines from white adipocytes, some of which are modulated by DNAm [101]. Adiponectin, an insulin-sensitizing and anti-inflammatory adipokine, has been implicated in longevity signaling pathways, including AMPK, SIRT1, and PGC-1α [61], which exhibits a low circulating levels in elderly individuals [102]. In fact, methylation in the *AdipoQ* promoter represses adiponectin expression in WAT [103]. Additionally, adiponectin is hypermethylated in the promoter region under obese conditions [28, 103-106]. Similarly, the peptide hormone leptin, which mediates adipose tissue-brain communication to reduce food intake and body weight [107, 108], is also essential for developmentally induced beige adipocyte formation and white adipocyte browning [109-111]. In fact, the function of adipose tissue declines as the progression of aging process, which is also coupled with the change of leptin level. In particular, the frail elderly is featured by the low circulating levels of leptin as compared to the healthy counterparts [112, 113], indicating that higher leptin levels may serve as a hallmark to prevent systematic senescence, in which adipose tissue senescence is a major characteristic feature. More importantly, the expression of leptin is tightly controlled by DNA methylation [114], supporting that DNA methylation affects adipocyte senescence. Taken together, these findings suggest that altered DNA methylation disorganizes adipogenesis and the synthesis of adipokines in white adipocytes, predisposing to the initiation and progression of aging process (Fig. 2).

## The implication of DNA methylation in the senescence of brown and beige adipocytes

In the former scenario, there is an explicit relevance between metabolic disease and the loss of metabolically active BAT with aging, while the molecular circadian mechanisms remain obscure due to the limitation of current detective technologies. Although great strides in research have demonstrated that the production of growth hormone, estrogens, and androgens may positively modulate the activity of BAT [115], the detailed cellular switches underlying these effects remain to be elucidated. Recent studies have revealed that DNAm events play a critical role in regulating thermogenic and developmental gene expression in beige and brown adipocytes.

Indeed, there is feasible evidence that the loss of BAT and decreases in the beiging ability of AT are likely linked to the altered DNAm. For example, PR domain containing 16 (PRDM16) is an essential transcription factor in regulating the differentiation and development of brown and beige adipocytes [116-118]. However, DNA demethylation of CpG islands in the *Prdm16* promoter, likely mediated by TET proteins, is required for its expression [119, 120], by which demethylation of the *Prdm16* promoter improves brown/beige adipogenic capacity and alleviates obesity in aged mice challenged with high-fat diet [121]. Similarly, DNA methylation may also regulate transcriptional changes in genes involved in beige and brown adipose development and thermogenic activity. BAT thermogenic function is impaired during aging as manifested by the loss of UCP1, a unique mitochondrial marker [122]. Specifically, DNAm regulates UCP1 expression by altering the methylation levels of the *Ucp1* regulatory sequence elements, and demethylation of the *Ucp1* enhancer augments its expression in brown adipose tissue [123]. In contrast, the receptor-interacting protein of 140 kDa (RIP140) recruits DNA methyltransferases into the site of the *Ucp1* enhancer, by which it promotes methylation of the *Ucp1* enhancer to silence its expression [124, 125]. Studies in our group further revealed that senescent brown or beige adipocytes are featured by the changes DNAm levels and/or patterns, coupled with an impaired capability for energy expenditure (unpublished data). Given that alterations in the gene expression profile induced by DNA methylation during aging may be the key node of age-related impairments and diseases, we therefore assume that age-related alteration in DNAm may directly cause changes in the transcriptional landscape in charge of thermogenic adipocyte regenerative capacity and mitochondrial energy metabolism (Fig. 2).

## DNA methylation in the senescence of adipose progenitor cells

Cellular senescence disrupts the dynamic balance in mesenchymal stem cells (MSCs), and thus impairing their therapeutic potential to differentiate into functional cells [126, 127]. Similarly, adipocytes developed from senescent preadipocytes exhibit reduced lipogenic capacity, resulting in glucose intolerance. Additionally, excessive accumulation of senescent progenitor cells in aging tissues triggers the generation of SASP pro-inflammatory cytokines, which strongly impedes the renewal and repair of injured tissues [66, 128-130]. Recent clinical trials have demonstrated a promising result that the senolytics, Dasatinib plus Quercetin (D?+?Q), directly eliminates senescent cells, which alleviates systemic SASP factors in human AT [131]. However, the mechanisms underlying age-related functional declines in preadipocytes are yet to be fully elucidated.

Comparative analysis of human MSCs from young and elderly donors revealed highly significant DNAm differences within the specific CpG sites of genes involved in cell differentiation [132]. Furthermore, the expression of genes relevant to differentiation including *C/ebp-α* and *Ppar-γ*, is decreased in preadipocytes with increasing age. A genome-wide analysis of DNA methylation of human preadipocytes compared to that of mature adipocytes revealed that 2,701 genes were hypomethylated and 1,070 genes were hypermethylated after adipocyte differentiation [133]. In fact, aging results in the deterioration of human AT-MSCs primarily through increasing DNAm levels, which induces oxidative stress and mitochondrial dysfunction [134-136]. Interestingly, L-carnitine efficiently decreases the percentage of senescent cells in AT-MSCs partially by modifying the methylation status of the human telomerase reverse transcriptase (*hTERT*) promoter region [137-139]. The *hTERT* promoter methylation status determines the expression of hTERT and telomerase activity [137, 140] (Fig. 2).

Age-related DNAm patterns also appear to be an important factor to maintain the healthy differentiation of adipose progenitor cells as well. In particular, a significant difference in terms of DNAm patterns was characterized between adipose progenitor cells originated from young and aged subjects as well as mice (unpublished data). The compromise in replication and differentiation of preadipocytes has been shown to drive insulin resistance by limiting plasticity of adipose tissue. However, considerable investigation is required to determine the molecular mechanisms associated with DNA methylation underlying the alterations in adipogenic phenotypes.

## Obesity dysregulates DNA methylation to promote the adipocyte aging process

Weight gain and the accumulation of adipose tissue increase the risk of a variety of age-related diseases, such as cardiovascular disease, and type 2 diabetes. Indeed, aging and obesity seem to share the same disease mechanisms, and one exacerbates the other [80, 141, 142]. An increased burden of senescent cells is observed in adipose tissue from obese individuals compared to lean age-matched controls [80]. In addition, subcutaneous adipose tissue from obese patients exhibits shorter telomeres [143]. Moreover, DNAm status displays specific tissue changes under the influence of age and obesity induced by high-calorie diets [92, 144]. It has been noted that peripheral blood from obese subjects is characterized by decreased DNA methylation compared to lean individuals during the course of aging [145]. This phenomenon could result from the limited energy intake coupled with the induction of DNMT1 expression, leading to de novo methylation [146].

Obesity affects genomic DNAm in AT, which then exacerbates the aging process of adipocytes and impairs their function [71, 86, 147-149]. Obesity aggravates senescence and dysfunction of adipocytes by modulating the methylation levels of genes in insulin secretion and energy metabolic pathways [150]. A higher potency to induce DNA hypermethylation in genes associated with energy expenditure than in genes relevant to energy storage has observed in epididymal WAT challenged by HFD [90]. For instance, CpG methylation of the *Plin1* promoter in adipocytes from obese women is higher than that in adipocytes from lean women [151]. Increased BMI is associated with increased methylation at the hypoxia inducible transcription factor (*Hif3A*) locus in human adipose tissue [152], while DNA methylation in intron 1 of *Cpt1α*, which encodes the enzyme carnitine palmitoyltransferase 1α, is inversely associated with waist circumference [153]. One reason for the age-related decrease in lipogenic activity is the lower expression of fatty acid synthase (FASN), an obligatory enzyme for the cellular synthesis of long-chain fatty acids [154, 155]. Methylation levels of *Fasn* in the promoter and first exon determine its expression. Furthermore, aging promotes adipose tissue as an energy sensor that is extremely susceptible to overnutrition, for which DNA methylation would provide a molecular link between aging and obesity.

## Conclusion and perspective

DNAm has now been recognized to play a critical role in the pathogenesis of age-related functional decline. Some CpG sites are highly correlated with aging, in which a small number of CpG sites are able to accurately predict the age of donors [86]. These DNAm sites have remarkably strong correlations with chronological age that could be predicted from the DNAm patterns of blood, saliva, buccal swab samples [156], glial cells and neurons [157]. Despite these advancements, the impact of DNAm on adipocyte aging process is yet to be fully elucidated, which deserves further investigations. Of note, other than DNAm, additional epigenetic mechanisms such as post-translational modification of histones or non-histone proteins are likely involved in the regulation of aging process as well. For example, the protein demethylase KDM1A maintains beige adipocytes by decreasing H3K9me2 levels to enhance PPAR-α expression [158].

There is convincing evidence that DNAm represents a bridge linking nutritional insults and genes, thereby being involved in developmental and senescent processes in AT. Due to the reversible nature of epigenetic programming, therapeutic interventions to reverse deleterious epigenetic changes would be promising to prevent relevant age-related metabolic disorders. In particular, restoration of the DNAm patterns in AT has been suggested to recover its thermogenesis properties during aging. Although we now have more understanding regarding the effects of DNAm on adipose aging, how environmental confounders, particularly overnutrition, induce global or gene-specific DNAm changes (e.g., methylation levels and/or patterns) and contribute to the initiation and progression of adipose dysfunction and aging remains largely unknown. It is indispensable to identify a consistent, reliable, and independent signature of DNA methylation across individuals of advanced age. Therefore, elucidation of the mechanisms underlying cellular senescence in adipocytes would have great potential for the development of approaches for increasing longevity and improving precision health.

Currently, the advancement of whole-genome methylation sequencing data would provide us the feasibility to identify comprehensive DNAm biomarkers for the adipose aging process and use such DNA methylation mechanisms for treatment or diagnosis of adipocyte senescence earlier. However, the critical issue is the development of approaches to modulate specific methylation machinery (e.g., modulating the methylation levels or patterns for a specific gene or a set of specific genes) that corresponds to the aging phenotype in adipocytes. Therefore, the development of gene-specific DNAm modulators and optimization of their dosages for the treatment of adipocyte senescence would be a major focus and worthwhile for future investigation. Likewise, DNA methyltransferase inhibitors, including azacitidine (5’-AZA) [159], decitabine [160, 161], zebularine [162, 163], and RG108 [161], have already been applied to treat various types of cancer, systemic sclerosis (SSc) and myelodysplastic syndrome (MDS) [164, 165]. However, severe side effects attenuated their application in clinical settings. In sharp contrast, the “readers” for DNA methylome (i.e., the methyl-CpG-binding domain proteins) are featured by the expression in a cell- or tissue-type dependent manner along with induction under diseased condition. More importantly, those MBD proteins themselves do not affect DNAm but interpret the effect of DNAm on target gene transcription [90, 166]. Particularly, MBD2 appears to be dispensable for biological processes under physiological condition[167-169]. Therefore, therapeutic strategies aimed at modulating the expression and/or functionality of DNAm readers, such as MBD2, could be a viable approach to prevent or treat adipocyte aging in clinical settings.
